# Supplementary material for: A Novel Mutation Associated with Neonatal Lethal Cardiomyopathy Leads to an Alternative Transcript Expression in the X-Linked Complex I NDUFB11 Gene
Source: Int J Mol Sci. 2023 Jan 16;24(2):1743. doi: 10.3390/ijms24021743 (PMC9865986; doi:10.3390/ijms24021743)
Supplement: Supplementary file 1 [file ijms-24-01743-s001.zip › ijms-2055091-supplementary.pdf]

Supplementary Material

A novel mutation associated with neonatal lethal cardiomyopathy leads to an alternative transcript expression in the X-linked complex I *NDUFB11* gene.

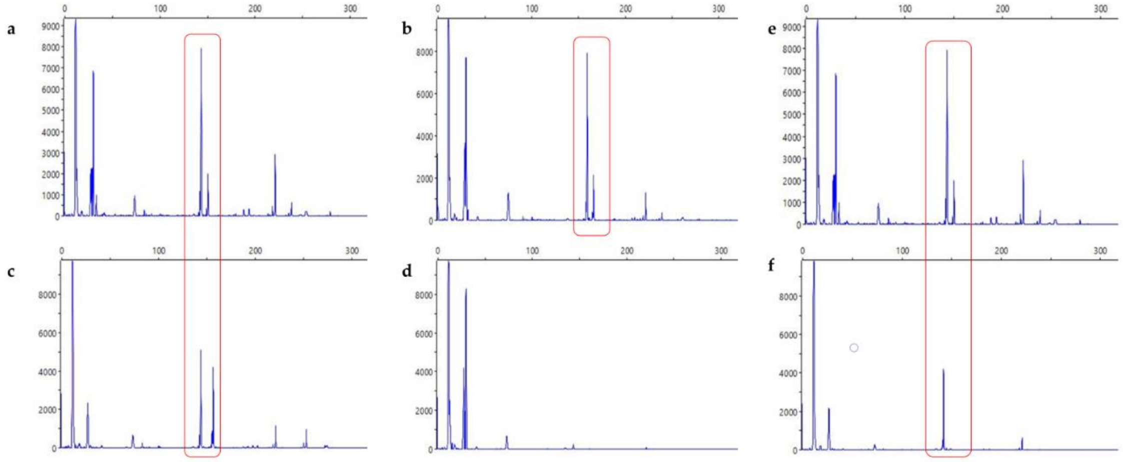

Supplementary Figure S1: X-chromosome inactivation (XCI) analysis by HUMARA test.

Panels show the DNA fragment patterns from proband's mother (S4a) and maternal aunt (S4b) obtained by capillary electrophoresis after methylation sensitive *Hpa*II restriction enzyme digestion. *Hpa*II recognize the active unmethylated allele, performed on total blood gDNA and subsequent PCR amplification of inactive methylated allele of exon 1 *human androgen receptor* gene (*HUMARA*). HUMARA test was performed in four noncarriers females as control for methylation status assay (S4c) (n=4), and three independent assays were performed. Complete digestion of unmethylated (active) X chromosome was tested by using a XY as a control (S4d). Panel S4e-f represents the peaks alignment between healthy mother (S4e) and proband's active allele (in absence of *Hpa*II) (S4f). Each digested sample data was normalized to undigested sample values to obtain % XCI. Results were expressed as the ratio of the preferentially inactivated allele.

Supplementary Table S1: Next-generation sequencing (NGS)-“OXPHOS” panel including 133 nuclear encoded structural subunits and assembly factors.

ACAD9	*ATP5J*	*COA1*	*COX18*	*COX7A1*	*LYRM7*	*NDUFA8*	*NDUFB11*	*NDUFS2*	*PET112*	*TMEM126B*
ATP5A1	*ATP5J2*	*COA3*	*COX19*	*COX7A2*	*NDUFA1*	*NDUFA9*	*NDUFB2*	*NDUFS3*	*SCO1*	*TMEM70*
ATP5B	*ATP5L*	*COA4*	*COX20*	*COX7B*	*NDUFA10*	*NDUFAB1*	*NDUFB3*	*NDUFS4*	*SCO2*	*TTC19*
ATP5C1	*ATP5L2*	*COA5*	*COX4I1*	*COX7B2*	*NDUFA11*	*NDUFAF1*	*NDUFB4*	*NDUFS5*	*SDHA*	*UQCC1*
ATP5D	*ATP5O*	*COA6*	*COX4I2*	*COX7C*	*NDUFA12*	*NDUFAF2*	*NDUFB5*	*NDUFS6*	*SDHAF1*	*UQCC2*
ATP5E	*ATP5S*	*COA7*	*COX5A*	*COX8A*	*NDUFA13*	*NDUFAF3*	*NDUFB6*	*NDUFS7*	*SDHAF2*	*UQCR10*
ATP5F1	*ATPAF1*	*COX10*	*COX5B*	*COX8C*	*NDUFA2*	*NDUFAF4*	*NDUFB7*	*NDUFS8*	*SDHB*	*UQCR11*
ATP5G1	*ATPAF2*	*COX11*	*COX6A1*	*CYC1*	*NDUFA3*	*NDUFAF5*	*NDUFB8*	*NDUFV1*	*SDHC*	*UQCRB*
ATP5G2	*ATPIF1*	*COX14*	*COX6A2*	*ECSIT*	*NDUFA4*	*NDUFAF6*	*NDUFB9*	*NDUFV2*	*SDHD*	*UQCRC1*
ATP5G3	*BCS1L*	*COX15*	*COX6B1*	*FASTKD2*	*NDUFA5*	*NDUFAF7*	*NDUFC1*	*NDUFV3*	*SURF1*	*UQCRC2*
ATP5H	*CMC1*	*COX16*	*COX6B2*	*FOXRED1*	*NDUFA6*	*NDUFB1*	*NDUFC2*	*NUBPL*	*TACO1*	*UQCRFS1*
ATP5I	*CMC2*	*COX17*	*COX6C*	*LRPPRC*	*NDUFA7*	*NDUFB10*	*NDUFS1*	*OXA1L*	*TIMMDC1*	*UQCRH*
										UQCRQ

The selection of the genes was performed in November 2015.

Supplementary Table S2: Primers sequence for *NDUFB11* gene and mRNA amplification.

Assay	Forward Primer (5′→3′)	Reverse Primer (5′→3′)
gDNA *NDUFB11* exon 2	ACCAAGTTATGCAGGGCCAGAATAA	TGGCAGAATCCCCTTCAACCTCCAT
cDNA *NDUFB11* exon 1-3	AACAACCAAGTCCGAGACTGGAGGC	CACTCATCCTCTGGCAGCTGGATCT
cDNA *NDUFB11* exon 2-3	AGGACGAAAACTTGTATGAGAAGAA	GTCGAAGCAGTTGGATTCCATGAT
canonical cDNA *NDUFB11* qPCR	GCCTGACTACAGGATGAAAGAG	GGGTCGAAGCAGTTGGATT
alternative cDNA *NDUFB11* qPCR	ACAGGGTGTCCAAGAGCGTG	CTTGCTGGGGTCGAAGCAGT
cDNA *HPRT1* qPCR	CCTGGCGTCGTGATTAGTGA	CGAGCAAGACGTTCAGTCCT

Supplementary Table S3. Thermocycling conditions pro *NDUFB11* gene amplification.

a)

	30 cycles				
94ºC/5′	94ºC/1′	58ºC/1′	72ºC/1′	72ºC/10′	4ºC/ ∞

b)

	40 cycles				
94ºC/5′	94ºC/45′′	60ºC/45′′	72ºC/45′′	72ºC/10′	4ºC/ ∞

c)

	30 cycles				
94ºC/5′	94ºC/45′′	60ºC/45′′	72ºC/45′′	72ºC/10′	4ºC/ ∞

a) Thermocycling conditions for *NDUFB11* (exon 1-3) with PCR amplification performed on gDNA. b) Thermocycling conditions for *NDUFB11* (exon 1-3) with PCR amplification performed on cDNA. c) Thermocycling conditions for *NDUFB11* (exon 2-3) with PCR amplification performed on cDNA.
